# Mindful Lawyering: a Pilot Study on Mindfulness Training for Law Students

**DOI:** 10.1007/s12671-022-01965-w

**Published:** 2022-08-19

**Authors:** Clifford J. Rosky, R. Lynae Roberts, Adam W. Hanley, Eric L. Garland

**Affiliations:** 1 S.J. Quinney College of Law, University of Utah, 383 S. University St, Salt Lake City, UT 84112 USA; 2 Center On Mindfulness and Integrative Health Intervention Development (C-MIIND), University of Utah, 395 South 1500 East, Salt Lake City, UT 84108 USA; 3 College of Social Work, University of Utah, Goodwill Humanitarian Building, 395 South 1500 East, Salt Lake City, UT 84108 USA

**Keywords:** Mindfulness, Meditation, Law, Well-being, Stress, Anxiety, Depression, Alcohol

## Abstract

**Objectives:**

Many US law schools are now offering elective courses in mindfulness training to alleviate disproportionately high levels of anxiety, depression, stress, and disordered alcohol use among law students. To date, empirical evidence on the effectiveness of these courses has been lacking. The aim of this pilot study was to explore the feasibility and impact of a 13‐week mindfulness course, “Mindful Lawyering,” specifically tailored to law students. The primary hypothesis was that mindfulness training would be significantly correlated with improvements in well-being and mindfulness.

**Methods:**

The design was a non-randomized, quasi-experimental study involving 64 law students. The mindfulness group was 31 students taking Mindful Lawyering; the comparison group was 33 students taking other law school courses. Outcome measures were the Depression, Anxiety, and Stress Scale; the Positive and Negative Affect Scale; the Alcohol Use Disorders Identification Test; and the Five Facet Mindfulness Questionnaire.

**Results:**

Results provide promising evidence to support the hypothesis. The mindfulness group showed significantly greater improvement on measures of stress (*p* < .001, *d* = 1.15), anxiety (*p* < .001, *d* = . 90), depression (*p* = .012, *d* = .66), negative affect (*p* = .002, *d* = .81), disordered alcohol use (*p* = .011, *d* = .67), and mindfulness (*p* < .001, *d* = 1.32) from pre to post relative to the comparison group. The course was well accepted and feasible for law students.

**Conclusions:**

Findings from the current study suggest that mindfulness training may occasion improvements in the well-being of law students. More research is needed to replicate these findings in larger, randomized samples of law students.

**Supplementary Information:**

The online version contains supplementary material available at 10.1007/s12671-022-01965-w.

For several decades, law students and lawyers in the USA have reported higher rates of anxiety, depression, stress, and disordered alcohol use than the general public (AALS, 1994; Beck et al., 1995; Benjamin et al., 1986, 1990; Dammeyer & Nunez, 1999; Eaton et al., 1990; Sheldon & Krieger, 2004). Within law schools, surveys have found that although students begin school with normal well-being measures, they experience significant declines during the first year of law school (Benjamin et al., 1986; Sheldon & Krieger, 2004).

Most recently, a survey of 3300 students from 15 US law schools found that 17% screened positive for depression, 37% for anxiety, and 43% for binge drinking in the prior 2 weeks (Organ et al., 2016). Another survey of more than 12,000 practicing attorneys in the USA found that 19% screened positive for anxiety, 28% for depression, 23% for stress, and 21% for problematic alcohol use (Krill et al., 2016). In this survey, problematic alcohol use was most prevalent among lawyers aged 30 or younger (32%, *p* < 0.001).

In response to these data, the American Bar Association (ABA) launched a National Task Force to address the well-being of law students and lawyers (National Task Force, 2017). The ABA’s report includes specific recommendations for how law schools, law firms, courts, and professional associations can build a “more sustainable culture” for students and lawyers.

One of these recommendations is mindfulness meditation. The ABA report cited meta-analytic evidence that mindfulness can reduce acute symptoms of anxiety and depression (Hofmann et al., 2010). Since the report was released, there have been additional studies and meta-analyses finding that mindfulness training can improve anxiety, depression, and stress (Blanck et al., 2018; Goldberg et al., 2019). In addition, several studies have indicated that mindfulness may be an effective treatment for disordered alcohol use (Bowen et al., 2006, 2007; Fernandez et al., 2010; Garland et al., 2010; Witkiewitz et al., 2005; Zgierska et al., 2008).

The ABA’s recommendation builds upon a growing movement to offer mindfulness training to law students and lawyers. Since the 1990s, an increasing number of judges, lawyers, law professors, and law students have begun to receive mindfulness training at a variety of retreat centers, law firms, and law schools across the USA (Riskin, 2002). Riskin (2002, p. 46) advocated for the expansion of these offerings, arguing that “mindfulness practice could (1) help lawyers and law students feel better and perform better at virtually any task; and (2) enable some lawyers to listen and negotiate better, thereby providing service that is more responsive to their clients’ needs.” Since then, many law professors have begun offering students mindfulness training in new elective courses and incorporating mindfulness training into the pedagogy of more traditional courses (Confino, 2019; Scott & Verhaeghen, 2020). In addition, many scholars have joined Riskin in calling for the widespread adoption of similar course offerings, claiming that mindfulness training can help students not only feel better but also perform better—reducing the anxiety, depression, and stress that many students experience during law school, while developing a wide range of essential lawyering skills (Brostoff, 2016; Confino, 2019; George, 2015; Harris, 2011; Huang, 2015; Iglesias, 2014; Rogers, 2015; Scott, 2018).

In recent years, mindfulness-based interventions have been studied in various higher education settings, including populations of graduate and professional students in medical school, nursing school, psychology, educational counseling, and other programs (Aherne et al., 2016; Barbosa et al., 2013; Barry et al., 2019; Beddoe & Murphy, 2004; Cohen & Miller, 2009; de Vibe et al., 2018; Ratanasiripong et al., 2015; Tarrasch, 2015). These findings of effectiveness among other graduate and professional student populations suggest that mindfulness may be beneficial for law students.

However, empirical evidence on how mindfulness training affects law students is still lacking. To date, remarkably few studies have been published on this subject, and the extant research has been published in journals that are not peer reviewed (Reuben & Sheldon, 2019; Scott & Verhaeghen, 2020). Reuben & Sheldon (2019) claimed that mindfulness training had statistically significant effects on stress, well-being, and mindfulness, but the study’s measure of well-being was not validated, and the study’s findings on stress and mindfulness were based on *p* values of 0.079 and 0.075, with no reported effect sizes. Scott & Verhaeghen (2020) found significant effects on depression (*p* < 0.05; *d* = 0.48), stress (*p* < 0.01; *d* = 0.70), negative affect (*p* < 0.05; *d* = 0.44), and mindfulness (*p* < 0.001; *d* = 1.11) in one of two samples, but these findings were limited by the study’s lack of a comparison group, the small size (*n* = 23) and low participation rate (21%) of the relevant sample, and a lack of correlations between the study’s findings on mindfulness and depression, stress, and negative affect. Neither study sought to determine how mindfulness training might affect disordered alcohol use among law students. While the findings from these studies are encouraging, further research is needed to confirm conclusions regarding the effects of mindfulness training on law student well-being.

The aim of this quasi-experimental study was to explore the feasibility and impact of a 13‐week mindfulness course specifically tailored to the needs and demands of law students. We hypothesized that mindfulness training would be associated with improvements in the well-being and mindfulness of law students.

## Method

### Participants

Participants were 64 out of the 179 (32%) second- and third-year students enrolled in S.J. Quinney College of Law at the University of Utah in Salt Lake City, UT, USA, between January and April 2020. Students were recruited to participate in the study via email. The mindfulness group was composed of 31 students who were taking an elective course called “Mindful Lawyering” along with other courses offered to second- and third-year law students. The comparison group was composed of 33 students who were not taking Mindful Lawyering but were taking other courses offered to second- and third-year law students.

The mindfulness group represented 31 out of the 45 (69%) students enrolled in Mindful Lawyering and other courses between January and April 2020. The comparison group represented 33 out of the 134 (25%) second- and third-year students enrolled exclusively in other courses during the same period. The study was limited to second- and third-year law students because Mindful Lawyering is an elective course, and first-year law students are not permitted to take elective courses. Three students were excluded from the comparison group because they had completed Mindful Lawyering in the previous academic year. Three students from the mindfulness group and five students from the comparison group completed the study’s procedures in January but not in April, yielding completion rates of 91% for the mindfulness group, 87% for the comparison group, and 89% for all participants. Participants were compensated with $60 gift cards after completing the study’s procedures in January and April.

Baseline demographic and sample characteristics are presented in Table 1. Chi-square analyses (categorical variables) and *t* tests (continuous variables) demonstrated that none of the demographic variables differed significantly between groups, indicating that the two groups were well matched across the measured characteristics. The Scale of Readiness for Self-Improvement (SRSI) did not statistically differ between groups, indicating that both groups were equally open to self-improvement (*p* = 0.664; see Table 1).

**Table 1:** Participant demographics and characteristics

	Mindfulness group (*n* = 31)	Comparison group (*n* = 33)	Difference *p* value
Age, *M (SD)*	29.4 (6.8)	27 (5.1)	.117
Sex, *n* (%)			.968
Female	13 (42%)	14 (42%)	
Male	18 (58%)	19 (58%)	
Identify as LGBT, *n* (%)			.592
Yes	1 (3%)	2 (6%)	
No	30 (97%)	31 (94%)	
Race, *n* (%)			.456
Latinx/Hispanic	2 (7%)	4 (12%)	
Asian/South Asian	0	1 (3%)	
Native Hawaiian/Pacific Islander	0	1 (3%)	
White/Caucasian	28 (90%)	26 (79%)	
Multiracial/other	1 (3%)	1 (3%)	
Highest education level, *n* (%)			.238
﻿ Bachelor’s degree	25 (81%)	30 (91%)	
Graduate degree	6 (19%)	3 (9%)	
Academic year			.632
Second	16 (52%)	19 (58%)	
Third	15 (48%)	14 (42%)	
Relationship status, *n* (%)			.804
Single	14 (45%)	16 (48.5%)	
Married or equivalent	15 (48%)	16 (48.5%)	
Divorced	2 (7%)	1 (3%)	
Annual family income, *n* (%)			.672
Under $25,000	11 (35.5%)	9 (27%)	
$25,000–$49,999	3 (9.7%)	6 (18%)	
$50,000–$74,999	8 (25.8%)	6 (18%)	
$75,000–$99,999	3 (9.7%)	2 (6%)	
$100,000–$199,000	4 (12.9%)	8 (24%)	
$200,000 or more	2 (6.5%)	1 (3%)	
Scale of Readiness for Self-Improvement (SRSI), *M (SD)*	56.3 (7.3)	55.3 (11.4)	.664

### Procedures

The design was a non-randomized, quasi-experimental study (Rubin & Babbie, 2017). Participants in both the mindfulness group and the comparison group were assessed two times: (1) shortly before the intervention began, at the beginning of the semester in January 2020, and (2) shortly after the intervention ended, at the end of the semester in April 2020. The latter time was scheduled to coincide with the exam period to explore the potential benefits of the intervention during a stressful period. To avoid potential experimenter bias effects, assessment measures were administered and collected by law school staff who were not involved in the design of the study or the intervention. All participants were assigned identification numbers. None of the investigators had access to the names of any participants.

The Mindful Lawyering intervention was offered to students as a pass/fail elective course. The course was modeled after similar mindfulness courses currently taught at other law schools. The course was taught weekly in 2-h sessions, for a total of 13 classes. The core of the program focused on training students in mindfulness meditation and helping them to establish a daily meditation practice. Participants received training in the following mindfulness practices: (1) “Focused Attention,” which involves directing attention to the physical sensations of in-and-out breathing, and returning attention to these sensations when the mind wanders; (2) “Body Scan,” which involves progressively moving attention through the body from the toes to the head while observing physical sensations; (3) “Open Monitoring,” which involves the observation of sensations, emotions, and thoughts, without letting oneself be carried away by them; and (4) “Compassion,” which involves cultivating a feeling of benevolence directed toward self and others. Starting the second week of class, students were asked to practice one of these forms of mindfulness meditation for at least 5 min each day, including weekends. Every 2 weeks, students were invited to meditate for an additional 5 min, until they reached a daily total of 20 min. Students were asked to keep a journal of how many minutes they meditated each day. They were encouraged to be honest when keeping these journals, and they were not graded on the number of days or the number of minutes that they recorded. Weekly, before each class, students were asked to complete reading assignments on mindfulness and the practice of law. During the semester, each student submitted a total of three short reflection papers, in which they were asked to notice sensations, emotions, and thoughts that arose while they were meditating, while they were reading the assignments, or while they were writing the paper itself.

The instructor was the first author (C.R.), who is both a law professor and a trained mindfulness facilitator. At the beginning of each class, the instructor led students through a 20-min meditation period and then invited students to share whatever sensations, emotions, or thoughts they noticed during the meditation period. At the end of each class, the instructor facilitated interpersonal mindfulness exercises, in which students practiced listening and speaking to each other while observing their own sensations, emotions, and thoughts. Additionally, during four of the thirteen class periods, the instructor invited local attorneys and judges to speak briefly with students about how to navigate the challenges of studying and practicing law while practicing mindfulness and maintaining well-being.

Because the study was conducted during the period between January and April 2020, it took place during the onset of the COVID-19 global pandemic. On March 18, 2020, Mindful Lawyering was shifted from an in-person to a live online format, along with all other courses taught at the university’s law school.

### Measures

Demographic measures (race/ethnicity, age, gender, sexual orientation, gender identity) were obtained at baseline. In addition, at both time points, participants were asked questions about whether they had previously engaged in mindfulness practices and the duration of these practices. The questionnaires and well-being measures were administered via Qualtrics. Additional measures were administered via Inquisit (2016), which will be reported in a separate paper.

#### Readiness for Self-Improvement

Participants completed the Scale of Readiness for Self-Improvement (SRSI, Cronbach’s *α* = 0.88, McDonald’s *ω* = 0.88), a validated rating scale consisting of 14 items, to provide a measure of readiness for self-improvement and care for one’s overall health (Zawadzka, 2014). Items are rated on a Likert scale ranging from 1 (“this doesn’t describe me at all”) to 5 (“this definitely describes me”). This measure was specifically included to explore the possibility that students who enrolled in the mindfulness intervention may have experienced changes in well-being or cognitive function because they were more motivated than those in the comparison group to improve themselves, rather than because of the intervention itself.

#### Depression, Anxiety, and Stress

Participants completed a short form of the Depression, Anxiety, and Stress Scale (DASS-21, Cronbach’s *α* = 0.93, McDonald’s *ω* = 0.93), a validated rating scale consisting of 21 items which provide separate scores of depression, anxiety, and stress (Lovibond & Lovibond, 1995). Participants indicated the degree to which each statement applied to them over the past week by specifying an answer on a 4-point Likert scale ranging from “Did not apply to me at all” to “Applied to me very much, or most of the time.”

#### Positive and Negative Affect

Participants completed a short form of the Positive and Negative Affect Scale (PANAS, Cronbach’s *α* = 0.85, McDonald’s *ω* = 0.78), a validated, 20-item rating scale that provides measures of both positive and negative affect (Watson et al., 1988). Participants responded to each item on a 5-point Likert scale ranging from “very slightly or not at all” to “extremely.”

#### Disordered Alcohol Use

Participants completed the Alcohol Use Disorders Identification Test (AUDIT, Cronbach’s *α* = 0.84, McDonald’s *ω* = 0.87), a validated rating scale consisting of 10 items measuring alcohol use behaviors, consumption, and alcohol-related issues (Babor et al., 1992). Participants responded to each item on a 5-point Likert scale ranging from “never” to “4 or more times a week.” A score of ≥ 8 is indicative of dangerous levels of alcohol use.

#### Mindfulness

Participants completed a short form of the Five Facet Mindfulness Questionnaire (FFMQ-15, Cronbach’s *α* = 0.77, McDonald’s *ω* = 0.87), a validated rating scale consisting of 15 items measuring a respondent’s dispositional tendency for mindfulness (Baer et al., 2012). Items are rated on a 5-point Likert scale, ranging from 1 (never or very rarely true) to 5 (very often or always true). The FFMQ provides an aggregate dispositional mindfulness score from five measured facets of mindfulness: observing, describing, acting with awareness, non-judging, and non-reactivity.

### Data Analyses

Data were analyzed with SPSS (version 27). Statistical significance was determined by an alpha < 0.05 for all hypothesis testing. For all outcome measures, 2 (time: pre, post) × 2 (group: mindfulness group, comparison group) repeated-measures ANOVAs were used—and post hoc tests as indicated—to determine whether significant differences were observed between the mindfulness and comparison groups and whether the outcomes differed between the two time points. Analyses of covariance (ANCOVA), controlling for baseline scores, were conducted as sensitivity analyses in the case of significant baseline differences between groups.

No variable was missing more than 3% data, and the balance of missing data was similar between the groups. The patterns of missing data, as well as a non-significant Little’s MCAR test (*χ*^2^ = 3.85, df = 68, *p* = 1.00), were consistent with data being missing completely at random.

## Results

At baseline, the groups showed no significant difference in their reports of current mindfulness practice. However, at time 2, there was a significant difference in current mindfulness practice, indicating that 87% of the mindfulness group were practicing mindfulness compared to 0% of the comparison group. At endpoint, participants in the mindfulness group reported practicing mindfulness meditation a mean of 5 (SD = 1.8) times per week. See Table 2 for rates of mindfulness practice for participants at both time points and practice duration details.

**Table 2:** Frequency and type of mindfulness practice for both groups, before and after the semester

	Mindfulness group	Comparison group	Difference *p* value
Time 1			
Current mindfulness meditation practice, *n* (%)			.138
Yes	2 (6.5%)	0	
No	29 (93.5%)	33 (100%)	
*If yes,* length of practice, *n (% of subset)*			
0–6 months	0	0	
6 months–1 year	0	0	
1–2 years	1 (50%)	0	
2–3 years	0	0	
3 or more years	1 (50%)	0	
*If yes,* days per week, *n (% of subset)*			
1–2	0	0	
3–4	0	0	
5–6	1 (50%)	0	
7 days	1 (50%)	0	
*If yes,* minutes per session, *n (% of subset)*			
Less than 15 min	1 (50%)	0	
15–30 min	1 (50%)	0	
Over 30 min	0	0	
Time 2			
Current Mindfulness Practice, *n (%)*			< .001**
Yes	27 (87%)	0	
No	4 (13%)	33 (100%)	
*If yes*, length of practice, *n (% of subset)*			
0–6 months	24 (88.9%)	0	
6 months–1 year	1 (3.7%)	0	
1–2 years	1 (3.7%)	0	
﻿ 3 or more years	1 (3.7%)	0	
*If yes,* days per week, *n (% of subset)*			
﻿ 1–2	3 (11%)	0	
﻿ 3–4	7 (26%)	0	
﻿ 5–6	9 (33%)	0	
﻿ 7 days	8 (30%)	0	
*If yes,* minutes per session, *n (% of subset)*			
﻿ Less than 15 min	18 (66.7%)	0	
﻿ 15–30 min	8 (29.6%)	0	
﻿ Over 30 min	1 (3.7%)	0	

^*^p < .05; **p < .001

**Fig. 1:**
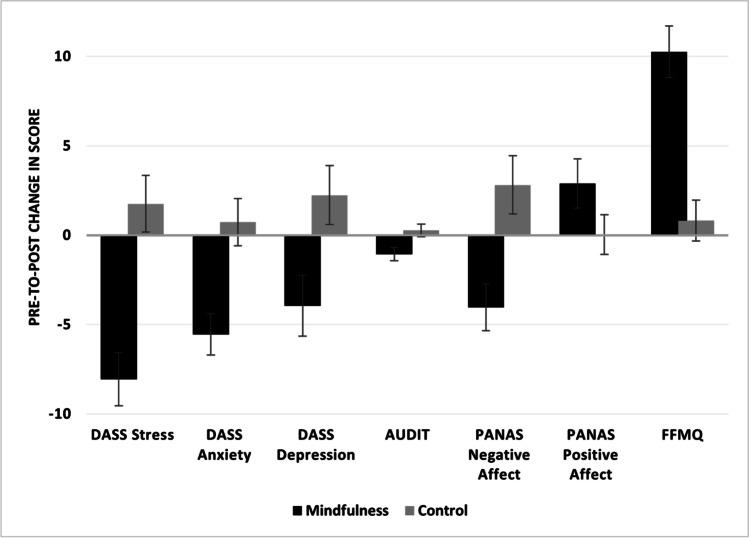
Pre to post change in outcome measures for each group. Error bars are standard error of the mean

See Table 3 for descriptive statistics for the self-report well-being questionnaires at both time points for both groups. The mindfulness and comparison groups were statistically equivalent on DASS depression scores, PANAS positive affect, and AUDIT scores at baseline. However, *t* tests demonstrated that between-group differences were present on the measures of stress (*p* < 0.001), anxiety (*p* = 0.039), negative affect (*p* = 0.002), and dispositional mindfulness (*p* = 0.005) at baseline. For the outcome variables with significant between-group differences at baseline, we conducted ANCOVAs, controlling for baseline scores, as sensitivity analyses to determine whether changes from pre to post were partially explained by baseline variation. Figure 1 shows the change from pre to post for both groups on each of the outcome measures.

**Table 3:** Descriptive statistics for questionnaires

	Mindfulness group *n* = 31 *M (SD)*		Change from baseline *p* val	Comparison group *n* = 33 *M (SD)*		Change from baseline *p* val
	Time 1	Time 2		Time 1	Time 2	
DASS Stress	19.7 (8.0)	11.7 (6.6)	< .001**	12.3 (7.6)	14.1 (9.3)	.276
DASS Anxiety	11.9 (8.5)	6.3 (5.4)	< .001**	7.9 (6.5)	8.6 (7.9)	.587
DASS Depression	11.9 (10.6)	8.0 (6.6)	.029*	8.0 (8.6)	10.2 (8.6)	.183
PANAS Positive Affect	25.7 (7.7)	28.6 (8.1)	.043*	26.7 (9.3)	26.7 (7.9)	.978
PANAS Negative Affect	20.7 (7.1)	16.7 (6.4)	.006*	15.2 (5.4)	18.1 (8.0)	.091
AUDIT	4.9 (5.0)	3.8 (3.6)	.008*	3.0 (4.3)	3.3 (4.7)	.440
FFMQ	41.2 (7.7)	51.5 (5.8)	< .001**	47.2 (8.6)	48.0 (8.1)	.476

*DASS* Depression Anxiety Stress Scales, *PANAS* Positive and Negative Affect Schedule, *SRSI* Scale of Readiness for Self-Improvement, *FFMQ* Five Facet Mindfulness Questionnaire total score

^*^p < .05; **p < .001

### Stress, Anxiety, and Depression

Significant time × group interactions were observed for the DASS stress subscale, (*F*_1, 62_ = 20.351, *p* < 0.001, *d* = 1.15), anxiety subscale (*F*_1, 62_ = 12.557, *p* < 0.001, *d* = 0.90), and depression subscale (*F*_1, 62_ = 6.771, *p* = 0.012, *d* = 0.66), indicating that, on average, the mindfulness group had significantly greater reduction in stress, anxiety, and depression symptoms than the comparison group. A sensitivity analysis adjusting for baseline differences in stress and anxiety did not substantively alter these results (*F*_1,61_ = 6.91, *p* = 0.011, *d* = 0.67 and *F*_1,61_ = 7.45, *p* = 0.008, *d* = 0.70, respectively). Group remained a significant predictor of baseline-adjusted stress and anxiety, with greater improvements in the mindfulness group.

### Positive and Negative Affect

There was a significant time × group interaction for negative affect as measured by the PANAS (*F*_1, 62_ = 10.276, *p* = 0.002, *d* = 0.81), indicating that, on average, the mindfulness group had significantly greater reductions in negative affect than the comparison group. However, a sensitivity analysis adjusting for baseline differences in negative affect found that group was not a significant predictor of baseline-adjusted negative affect (*p* = 0.162). The time × group interaction was non-significant for the PANAS positive affect scale.

### Disordered Alcohol Use

There were significant time × group interactions on the AUDIT outcomes (*F*_1, 62_ = 6.858, *p* = 0.011, *d* = 0.67), indicating that, on average, those in the mindfulness course had significantly greater reductions in alcohol misuse behaviors and alcohol-related problems than the comparison group.

### Mindfulness

There was a significant time × group interaction on FFMQ total scores (*F*_1, 62_ = 26.803, *p* < 0.001, *d* = 1.32), indicating that, on average, the mindfulness group had significantly greater increases in the tendency to be mindful in everyday life than the comparison group. A sensitivity analysis adjusting for baseline differences in FFMQ did not alter these results (*F*_1,61_ = 14.74, *p* < 0.001, *d* = 0.99). Group remained a significant predictor of baseline-adjusted FFMQ scores, with greater increases in the mindfulness group.

### Correlates of Dispositional Mindfulness and Study Outcomes

We observed significant negative correlations between increased levels of mindfulness and decreased levels of anxiety (*r* =  − 0.57, *p* < 0.001), depression (*r* =  − 0.55, *p* = 0.001), negative affect (*r* =  − 0.51, *p* = 0.003), and disordered alcohol use measures (*r* =  − 0.36, *p* = 0.047) in the mindfulness group.

## Discussion

There were significant decreases in anxiety, depression, stress, and disordered alcohol use, with effect sizes ranging from moderate to large. In addition, the Mindful Lawyering course was also associated with significant increases in dispositional mindfulness, which were in turn correlated with improvements in the aforementioned measures. These findings are consistent with meta-analyses of how mindfulness affects depression, anxiety, and stress (Blanck et al., 2018; Goldberg et al., 2019; Hofmann et al., 2010). In addition, they are consistent with similar studies on how mindfulness training affects graduate and professional students (Aherne et al., 2016; Barbosa et al., 2013; Barry et al., 2019; Beddoe & Murphy, 2004; Cohen & Miller, 2009; de Vibe et al., 2018; Ratanasiripong et al., 2015; Reuben & Sheldon, 2019; Scott & Verhaeghen, 2020; Tarrasch, 2015). They provide promising evidence that the well-being and mindfulness of law students may be improved by integrating mindfulness training into law school curricula.

In particular, these findings replicate and strengthen several findings from Scott & Verhaeghen’s, 2020 study of law students at Georgia State University College of Law. In this quasi-experimental, non-randomized study, the authors recruited a total of 30 mostly first-year law students to complete pre-test and post-test surveys measuring the effects of two 6-week training programs conducted in fall 2018 and 2019, and one 7-week training program in summer 2018. Scott and Verhaeghen administered a wide array of measures, including the DASS, PANAS, and FFMQ, among several others. In the two fall programs (*n* = 23), the authors found significant decreases in depression, stress, and negative affect, and a significant increase in mindfulness. The present study’s inclusion of a comparison group and larger sample sizes bolsters the findings from this earlier work.

This study’s observed decrease in AUDIT scores is especially notable, in light of the prevalence of disordered alcohol use among law students and young lawyers. There is increasing evidence that mindfulness can help address a wide range of addictive disorders, including alcohol and substance use disorders, and some evidence for reducing alcohol craving and misuse (Garland et al., 2010, 2016, 2022; Korecki et al., 2020; Li et al., 2017; Mermelstein & Garske, 2015). For many years, studies have found elevated risks of alcohol use disorders among law students and lawyers (AALS, 1994; Beck et al., 1995; Benjamin et al., 1986, 1990; Dammeyer & Nunez, 1999; Eaton et al., 1990; Krill et al., 2016; Organ et al., 2016; Sheldon & Krieger, 2004). This pilot study suggests that mindfulness training may be an effective way to address and prevent disordered alcohol use among law students. During the course of the semester, the mindfulness group’s average AUDIT scores decreased by 22%, while that of the comparison group increased by 9%.

### Limitations and Future Research

The current study has several limitations. First, because the study was not randomized, for practical and logistical reasons, we were not able to control for group differences, and we cannot draw any firm causal inferences. Future studies should employ a randomized controlled design to rule out potential threats to internal validity.

The mean scores for the two groups across some measures suggest that there were some significant differences between the two groups from the outset. At time 1, the mindfulness group exhibited significantly higher levels of anxiety, stress, and negative affect than the comparison group, and significantly lower levels of mindfulness. This may be evidence of a self-selection bias: Students may have enrolled in Mindful Lawyering because they were experiencing more anxiety, stress, and negative affect than other law students, and may have therefore been more motivated to seek the benefits of establishing a mindfulness meditation practice.

It is important to note, however, that the two groups did not exhibit any significant differences on the Scale of Readiness for Self-Improvement. This data suggests that the practice of mindfulness meditation, rather than motivation for self-improvement, likely accounts for the difference in outcomes. Moreover, ANCOVAs found that Mindful Lawyering was associated with greater improvement on all of these metrics even after controlling for baseline differences. Although the mindfulness group began the semester with “mild” depression, “moderate” anxiety, and “moderate” stress, they ended the semester in the “normal” range on all items (Lovibond & Lovibond, 1995). In particular, the large effect size (exceeding one standard deviation) on stress in the mindfulness group suggests that participation in the course may be especially helpful in coping and managing with stress. The fact that the comparison group displayed an increase in stress, while the mindfulness group displayed such a large decrease, suggests that this effect may not be an artifact or a temporal effect.

Another limitation of this study is that the principal investigator both recruited students into the study and served as the instructor for Mindful Lawyering, the intervention itself. As a result, it is possible that the mindfulness group reported differences in anxiety, depression, and stress in order to please the principal investigator, rather than as accurate measures of the underlying emotions and thoughts. This risk was mitigated by having staff collect data, using confidential participant codes, and explaining that none of the investigators would ever learn which students participated. In future studies, it would be useful to rely on mindfulness instructors who are not investigators. Additionally, this study relied on a single instructor. As a result, it is possible that the mindfulness group reported differences related to the specific techniques of the instructor, rather than the intervention itself. In future studies, it would be useful to rely on multiple instructors, to eliminate any effects caused by a particular instructor.

Most of this study’s statistically significant findings are based on self-reported data which are subject to inherent bias. If students signed up for Mindful Lawyering for the purpose of reducing anxiety, depression, and stress by increasing mindfulness, they may have been telling themselves what they wanted to hear. In future studies, it would be useful to gather data by using objective measures, in addition to gathering self-reported data.

This study did not seek to measure the relationship between mindfulness and other variables, such as self-compassion and resilience, which may also improve the well-being of law students. Previous research has found significant correlations between mindfulness and these variables (Barry et al., 2019; Galante et al., 2018; Garcia et al., 2022). In future studies, it would be useful to gather data on the relationship between mindfulness and potentially protective variables.

The final limitation of this study is that it took place during the onset of the global COVID-19 pandemic. Such an unexpected development cannot be anticipated or addressed in future studies. Regardless, the fact that the mindfulness group showed significant improvements in validated measures of anxiety, depression, stress, negative affect, and alcohol use—even as they were experiencing the onset of a global pandemic—can be interpreted as an encouraging sign for the effectiveness of mindfulness education in law schools.

## Supplementary Information

Below is the link to the electronic supplementary material.

Supplementary file1 (XLSX 26 KB)

## Data Availability

All data are available at the Open Science Framework (https:// osf.io/qntp4).
